# Virtual Reality Simulation for Orthopedic Surgical Training: A Narrative Review of Current Evidence and Educational Impact

**DOI:** 10.7759/cureus.99367

**Published:** 2025-12-16

**Authors:** Ali Soffar, Ahmed Elkohail, Mohamed Elbanna, Hassan A Kassem, Siddhartha Murhekar, Mohammed S Millat, Abdelrahman Sayed, Rafat Shehata, Moaaz Ali Hamoud, Nervana Khalil

**Affiliations:** 1 Trauma and Orthopedics, Princess Royal University Hospital and Orpington Hospital, King's College NHS Foundation Trust, London, GBR; 2 Trauma and Orthopedics, Princess Royal University Hospital, King's College NHS Foundation Trust, London, GBR; 3 Trauma and Orthopedics, Epsom and St Helier University Hospitals, London, GBR; 4 General Internal Medicine, King’s College London, London, GBR; 5 Trauma and Orthopedics, Medway NHS Foundation Trust, Gillingham, GBR; 6 Trauma and Orthopedics, Prince Charles Hospital, Cardiff, GBR; 7 Emergency Medicine, Hampshire Hospitals NHS Foundation Trust, Basingstoke, GBR; 8 Orthopedic Surgery, Faculty of Medicine, Kafrelsheikh University, Kafrelsheikh, EGY; 9 Pediatric Surgery, Mansoura University Children Hospital, Mansoura, EGY

**Keywords:** arthroscopy, bone trauma, haptic feedback, medical education, orthopedic surgery, rehabilitation, simulation, surgical training, total hip replacement, virtual reality (vr)

## Abstract

Virtual reality (VR) simulation represents a significant advancement in orthopedic surgical training, offering a powerful supplement to traditional educational paradigms. This review highlights robust evidence demonstrating that VR is an excellent educational tool, capable of bridging the gaps left by reduced work hours and ethical considerations associated with traditional clinical training. A recent meta-analysis confirms that VR interventions significantly improve trainees' knowledge scores, clinical operation scores, and surgical design capabilities compared to conventional methods. This enhanced level of competency, dexterity, and spatial awareness is evident across diverse applications, including arthroscopy, total hip replacement planning, and trauma fixation. VR's ability to provide a safe, repeatable, and accessible learning environment facilitates skill acquisition that has shown transferability to the operating room. Despite these benefits, challenges remain, including technical limitations like system latency, difficulties with depth awareness, and the potential for cognitive overload or inattentional blindness. Crucially, many existing orthopedic trauma simulators require rigorous validation of their concurrent and transfer validity to ensure they meet the standards of established training models. Future advancements are expected to integrate biomechanical analysis for fracture repair and artificial intelligence to enhance intraoperative decision-making. As these technologies mature and undergo validation according to standardized guidelines, VR is poised to become an indispensable component of orthopedic education, improving surgical accuracy and patient outcomes.

## Introduction and background

In the past, the traditional educational paradigm has been essential to the spread of knowledge and the improvement of skills. Nevertheless, it has a number of difficulties, such as improving student participation in educational activities and successfully meeting their unique learning requirements [1,2]. Surgical simulators are used for training skills in numerous professions to generate an approximately realistic learning environment without the risk of patient damage. Although cadavers have long been considered the gold standard for instruction, there are drawbacks, including high expenses, restricted accessibility, non-pathologic states, and ethical issues. As a result, new surgical training possibilities have been investigated [3,4].

Virtual reality (VR) training has emerged as a revolutionary instrument with many benefits for clinical practice and in all medical fields [5]. Because VR simulations can highlight complex anatomical features and surgical techniques, students can engage with and control virtual models in real time. This capacity greatly improves the retention of theoretical information and the development of practical abilities [6,7].

VR technologies are currently being integrated into orthopedic training simulators. This integration aims to enhance surgical accuracy, ultimately leading to improved patient outcomes and a reduction in potential complications associated with orthopedic procedures [8-11]. VR devices are frequently used in orthopedic trauma operations, particularly for preoperative planning and fracture fixation. Studies indicate that by minimizing the need for fluoroscopic guiding, VR simulations may enhance intraoperative fracture reduction efficiency, improving surgical performance and lowering radiation exposure [12]. In this narrative review, we aim to determine the current evidence for the use of VR simulation in orthopedic surgical training and its educational impact.

## Review

Overview of VR in orthopedic education

Developing complete orthopedic competence solely within the clinical setting is no longer practical. The increasing cost associated with resident education, along with reduced time spent at work by residents and ethical considerations regarding patient well-being, necessitates that trainees acquire essential skills outside of the operating room [8,13].

Because of its wide range of applications, including bone cancers, sports injuries, joint disorders, and trauma, orthopedics poses many difficulties. Due to their complexity and connections to fields such as anatomy, radiography, and biomechanics, these subjects can be challenging to comprehend and remember [14]. Orthopedic surgery requires a deep understanding of complex anatomy and exact surgical methods. However, traditional training, which includes cadaver dissection, observing surgeries, and using simulation models, may not significantly improve orthopedic surgeons' academic results or clinical skills [15,16].

By offering a substitute for extra procedural instruction, VR can bridge these gaps in the current residency curriculum. With tools for performance analysis and recording, the technology may produce a realistic and highly dynamic operational simulation. Furthermore, these simulators are easily accessible for use without requiring a skilled instructor to supervise and advise the student. A study on the usage of the Minimally Invasive Surgical Trainer (MIST)-VR laparoscopic simulator found that residents who have received VR training do surgery more quickly, while residents who do not have access to VR are more likely to inadvertently injure themselves [17].

VR offers the most advanced and validated reality technology for surgical training, immersing users in a completely computer-generated environment. Its current applications are limited to education and preoperative planning. While arthroscopy techniques have more readily available simulators, VR training hardware and software exist for a variety of both open and arthroscopic procedures. Importantly, VR simulators have demonstrated greater effectiveness in enhancing resident learning compared to synthetic models or benchtop trainers [18,19].

The majority of VR training programs on the market today use haptic device technology, which applies force feedback to the user's hand to enable the user to feel computer-generated surgical instruments and anatomy on screen. Even lower frequency vibration feedback can be provided using Touch 3D Systems technology to replicate drilling forces and torques [10].

Benefits of VR applications in orthopedic surgery

Orthopedic trainees' procedural competence and practical skills are improved by VR instruction. The improved surgical design and clinical operation ratings demonstrate how well VR simulations support practical training and skill improvement. In a secure and encouraging setting, VR helps trainees to improve their muscle memory, spatial awareness, and surgical dexterity by enabling repeated and controlled simulations of surgical procedures [20,21]. VR has revolutionized orthopedic surgery by improving preoperative planning, intraoperative navigation, and postoperative rehabilitation. These VR technologies enhance surgical accuracy, reduce operating time, and lead to better patient outcomes through interactive and immersive visualization tools [22].

**Figure 1:**
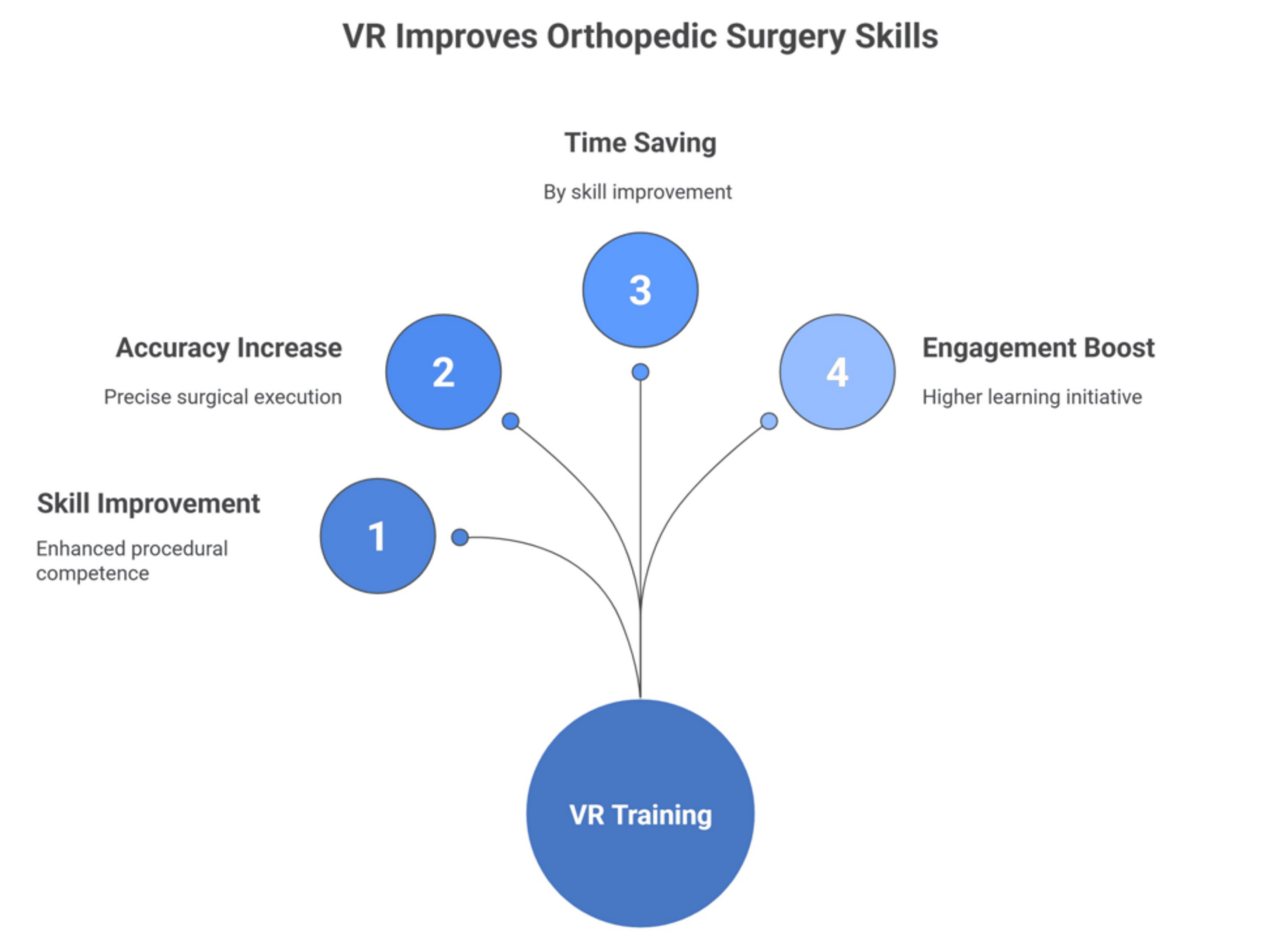
Benefits of VR Applications in Orthopedic Surgery Figure created by the article authors based on references [19-23]. VR: virtual reality

Li et al. conducted a systematic review and meta-analysis to evaluate the effectiveness of VR compared to traditional education for orthopedic surgical training. The analysis included 23 randomized controlled trials involving 1,091 participants. The researchers searched databases up to July 2024, comparing outcomes across knowledge, practical skills, and student engagement. The results demonstrated that VR interventions led to significantly superior outcomes. Participants in VR groups achieved higher knowledge scores, clinical operation scores, surgical design scores, clinical understanding, and clinical thinking ability compared to those receiving traditional education. Furthermore, VR-based learning was associated with markedly higher levels of teaching interest, satisfaction, and greater initiative in learning. The authors conclude that VR is an excellent educational tool in orthopedics, significantly boosting both theoretical knowledge and practical skills, as well as student engagement. However, they note the need for further high-quality, large-scale trials (Figure 1) [23].

Total Hip Replacement

Sato et al. created simulations of the total hip replacement (THR) preoperative phase. This system makes it possible to prepare for the best possible selection of factors, including stem orientation, position, and cup size. These are ascertained through surgical planning that incorporates limb length correction and range of motion (ROM) simulation. Also, a three-dimensional (3D) preoperative hip implant planning tool created by Jun and Park analyzes each patient's unique 3D hip anatomy to produce a prosthesis tailored to their needs. Following a 3D reconstruction of the hip from CT scans, a customized hip prosthesis is designed for the patient, a virtual femoral head resection and simulated implantation are carried out, the prosthesis' fit and functionality are assessed, and 3D surgical parameters are generated to direct the actual procedure [8].

Dick et al. created a 3D computer simulation tool for preoperative hip implant planning. This tool utilizes patient-specific CT scan finite element data to mimic how a patient's bone will react mechanically to an implant load. The goal is to identify the optimal implant design and size that produces the most natural stress distribution [24].

Sawbones created a manikin-based hip arthroscopy bench-top simulator. A study by Pollard et al. showed that this simulator can objectively improve trainee learning. Trainees are likely to benefit from simulator training to achieve basic competence in both lateral and supine orientations. However, the Sawbones simulator, like other manikin simulators, is only a bench-top model and does not utilize VR technologies [25].

A computer-driven system has been designed to assist surgeons during revision THR surgeries. This simulator aims to decrease the time and effort needed for cement removal, avert complications like cortical wall penetration and femoral fracture, improve the placement and fit of the new implant, allow for accurate and high-quality canal milling, and minimize the amount of bone that must be removed for the new implant [26].

The Robodoc is a computer-integrated surgical robot, introduced in 1990 by Integrated Surgical Systems (ISS) and FDA-approved in 1997. It was designed to improve THR by minimizing human error through its five-axis robotic arm with a milling device. The first human hip surgery using Robodoc occurred in 1992. The system includes Orthodoc, a preoperative planning workstation that uses CT scans to create 3D bone images and model various implants [27,28].

Arthroscopic Surgery Training

Arthroscopy is a flexible orthopedic procedure for diagnosis and treatment that involves combining fundamental skills to execute intricate operations. It necessitates two-handed coordination and 3D triangulation, guided by a 2D intra-articular video feed [29]. This can be difficult and requires a significant amount of time to master [30,31].

Strong evidence indicates that VR arthroscopic simulators can distinguish between surgeons of varying experience levels (novice, intermediate, and expert) in shoulder and knee arthroscopy, and also enhance skills such as efficient movement, camera control, and triangulation [32-37]. VR simulator training not only enhances performance on cadaveric arthroscopic models but also has shown transferability to real-world operating room settings [30,38-40].

Cannon et al. evaluated the construct validity of the ArthroSim VR arthroscopic knee simulator by testing whether surgical experience translates to better simulator performance. Six PGY-1 orthopedic residents, six PGY-5 residents, and six community orthopedic surgeons used the simulator to perform a diagnostic knee arthroscopy. Outcomes were procedure time and a completeness score reflecting the correct sequence of steps. Mean times were 1,028 s for PGY-1 residents, 745 s for PGY-5 residents, and 610 s for practicing surgeons; senior residents and surgeons were significantly faster than PGY-1 residents (p = 0.006). Completeness scores were similar (71%, 79%, and 85%) and not significantly different, suggesting acceptable consistency. Faster performance did not reduce accuracy. The authors concluded that greater clinical experience improves efficiency on the simulator and noted future training modules for meniscectomy and meniscal repair [33].

Bone Trauma 

Orthopedic training and practice face a growing challenge, concurrent with the increasing prevalence of traumatic bone conditions. This is due to current trends in clinical work hours, healthcare budgets, legal frameworks, patient safety concerns, and public expectations, all of which significantly influence surgical training and practice [41-43]. A troubling decrease in the number of orthopedic and trauma specialists has been consistently observed and thoroughly documented over the last several decades [44-47].

A global study utilizing the Global Burden of Disease (GBD) framework revealed a significant increase in fracture incidents, with 178 million reported in 2019. This figure represents a substantial 33.4% growth in fracture occurrences since 1990, highlighting a growing global health concern [48]. Annually, approximately 340,000 hip fractures are reported in elderly patients in the United States, while Europe saw around 600,000 hip fractures in 2010 [49-52].

With advances in computing and imaging, various reality technologies have emerged for clinical and educational use. In orthopedic trauma surgery, VR, augmented reality (AR), and mixed reality are employed for teaching, practice, and patient-specific simulations. These are sometimes grouped under the term "extended reality" [53].

Fixing bone fractures requires carefully putting the broken pieces back together and securing them with screws, plates, or other implants, depending on the type and complexity of the break. Accurate execution is crucial for creating the best conditions for the bone to heal and regain its original shape and function. If a joint like the hip, knee, or ankle is involved, precise repair of the joint surface is vital to prevent arthritis later on. Greater surgical precision in orthopedic trauma leads to safer procedures, proper anatomical healing, and fewer postoperative problems and long-term issues such as delayed, improper, or failed healing and posttraumatic arthritis [54-57].

TraumaVision, a software created by Swemac, Melerit, and Simulation Inc., offers simulations for various orthopedic trauma cases, including fractures of the femoral neck, trochanter, subtrochanter, and femoral shaft, as well as pelvis and spinal surgeries, slipped capital femoral epiphysis, and Motec^®^ wrist prosthesis procedures. The software includes training modules for drill skills, cannulated screws, dynamic hip screws, and fluoroscopy. Fluoroscopy images (conventional A-P and lateral radiographs) are captured by pressing a foot pedal. A computer-connected robot arm, Phantom Omni, can be controlled by either hand to simulate surgical tools and provide haptic feedback [58].

The BoneDoc DHS simulator is a web-based VR system that allows users to practice hip fracture fixation surgery on a standard computer with a mouse. It leads users through the surgical process, from reducing the fracture to inserting the implant, using 2D X-rays and a 3D hip model. Although it does not offer haptic feedback, the simulator provides assessment and has been shown to be realistic, accurately replicating the actual surgery [59,60].

LeBlanc et al. compared orthopedic resident performance in ulnar fracture fixation using a new haptic VR simulator versus a traditional Sawbones model. Twenty-two residents were randomized to use one simulator, then the other. Both simulators successfully distinguished between different resident experience levels, showing construct validity. Participants generally scored significantly better on the VR simulator for most measures, except for the time taken. However, there was no significant correlation between resident scores on the two different simulators, meaning concurrent validity was not established. The authors concluded that the VR simulator shows promise for training [61].

Musculoskeletal Rehabilitation

By integrating telemedicine with wearable technology, extended reality (XR) rehabilitation facilitates the delivery of musculoskeletal care beyond the confines of conventional clinical settings. Central to these AR/VR recovery programs are inertial measurement units (IMUs), which offer continuous and objective monitoring of joint kinematics and gait. When affixed to the limbs, these sensors precisely capture motion data that can be digitally transmitted to healthcare providers in real time. Furthermore, recent studies confirm that modern IMU systems achieve an accuracy for knee motion metrics comparable to laboratory-based motion capture, while simultaneously permitting assessments in real-world environments [62].

The integration of VR into rehabilitation significantly boosts patient compliance and recovery by offering dynamic and engaging sessions. VR utilizes head-mounted displays (HMDs) to create a fully immersive digital experience that completely replaces the real world. In contrast, AR overlays digital materials onto the user's physical environment. This allows patients to see both their actual surroundings and virtual assistance simultaneously, using devices like smart glasses or tablets. This AR capability is particularly useful for providing real-time instruction and feedback during exercises. According to a comprehensive review, this AR-based approach, which facilitates guided and interactive movement therapy, shows significant potential for enhancing postinjury functional outcomes [63].

VR rehabilitation programs are highly effective for patients with musculoskeletal injuries and those recovering from joint replacement surgery. Research shows that these gamified environments lessen pain, increase ROM, and improve functional recovery by encouraging patients to actively participate [64].

VR-assisted rehabilitation has also been successfully implemented in the treatment of chronic musculoskeletal disorders, such as low back pain. Studies have consistently demonstrated its effectiveness in significantly reducing both pain-related fear and disability scores in affected individuals [65].

Providing therapy remotely through VR platforms preserves continuity of care, especially for patients hindered by logistical barriers to in-person rehabilitation, such as geographical distance, limited mobility, or scheduling conflicts. This remote accessibility allows for consistent therapeutic engagement and progress, overcoming common challenges that might otherwise disrupt treatment plans [66].

VR-based applications have demonstrated significant clinical benefits in various rehabilitation programs, extending beyond orthopedic training. For instance, these applications have been successfully utilized in reducing acute procedural pain experienced by patients during hand therapy sessions, thereby improving their overall experience and potentially enhancing treatment adherence [67].

Limitations and future directions

Any type of AR has decreased the time required to complete a procedure, but there are some limitations. One such issue that must be addressed when using 3D overlays is inattentional blindness, which occurs when a surgeon fails to notice an unexpected object that suddenly enters his field of vision [68].

The growing volume of information presented to surgeons via AR during surgical procedures raises concerns about potential distractions that could negatively impact their focus and performance [69]. As a result, it is essential to present only crucial information or offer a way to toggle between various data sets as needed.

Hansen et al. suggested a method to decrease cognitive demands involving optimizing the spatial arrangement of individual structures, minimizing AR-induced occlusion, and maximizing contrast, which allowed surgeons to lessen visual clutter in certain situations [70].

Achieving precise 3D and depth awareness still presents difficulties. Another problem is system latency, which can impair surgeon comfort and accuracy. For example, Kang et al. discovered that the delay of their optical system for laparoscopic surgeries was 144 ± 19 ms [71].

VR systems improve intraoperative and postoperative outcomes, in addition to facilitating procedural learning and enhancing preoperative planning. Integrating biomechanical analysis into VR preoperative planning for fracture repair could further enhance clinical outcomes [72]. AR/VR technology is expected to advance in orthopedics in the future, resulting in better rehabilitation techniques, patient-specific preoperative planning, and more accurate surgical guidance. Artificial intelligence-powered VR simulations have the potential to improve intraoperative decision-making and procedural planning in arthroplasty [63].

**Figure 2:**
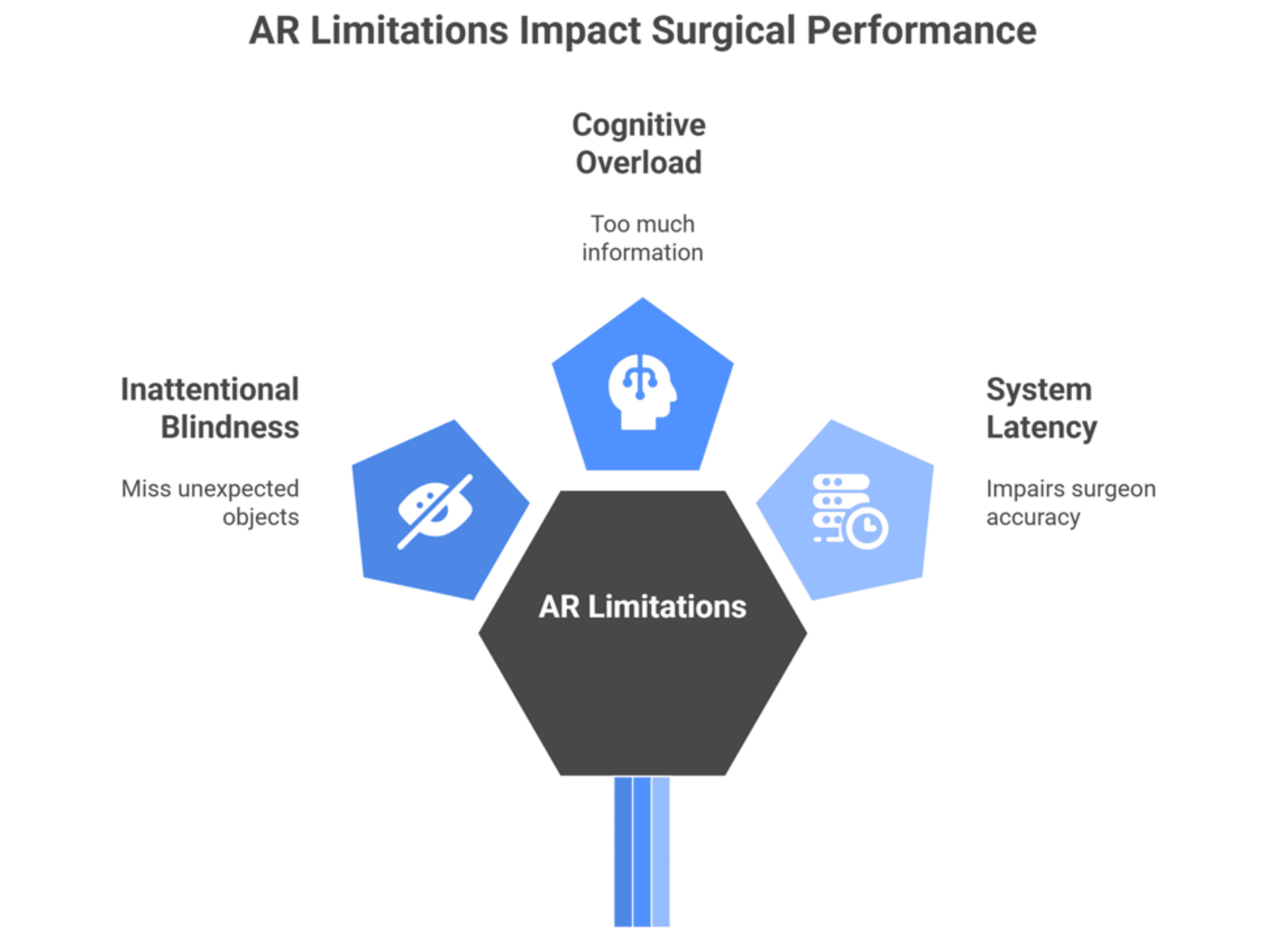
Limitations and Future Directions of the Use of AR in Orthopedic Surgery Figure created by the article authors based on references [68-73]. AR: augmented reality

Existing orthopedic trauma VR systems require rigorous validation of their face, content, concurrent, and transfer validity. Further development should align with industry standards for immersion, multisensory realism, and versatility. The European Association of Endoscopic Surgeons Work Group's guidelines for evaluating and implementing simulators and skills training programs offer a standardized approach for appraising simulation studies (Figure 2) [73].

## Conclusions

The landscape of orthopedic surgical training is fundamentally evolving, driven by the need to develop proficient surgeons in an environment constrained by reduced resident work hours, high costs, and paramount patient safety concerns. VR simulation is an excellent and effective educational tool to bridge these contemporary training gaps. VR training has emerged as a revolutionary instrument in orthopedics, offering realistic, accessible, and dynamic simulations that allow trainees to develop skills in a safe environment without risk of patient harm. VR simulation effectively improves essential surgical competencies, including spatial awareness, muscle memory, and dexterity. Crucially, skills developed on VR simulators, particularly in complex procedures like arthroscopy, have been shown to transfer to the operating room, leading to improved real-world surgical performance.

The application of VR spans the breadth of orthopedic practice. It is used extensively for preoperative planning in THR and for fracture fixation in trauma cases. It also serves as a key tool for intraoperative navigation and has transformed postoperative rehabilitation by improving patient compliance and functional recovery. While limitations such as system latency and the potential for cognitive distraction must be addressed, the future of VR in orthopedics is promising. Future directions should focus on the rigorous validation of new systems and the integration of artificial intelligence to further enhance procedural planning and intraoperative decision-making, ultimately improving surgical accuracy and patient outcomes.
